# Maintaining the Quality and Nutritional Integrity of Chilled *Cordyceps sinensis*: Comparative Effects and Mechanisms of Modified Atmosphere Packaging and UV-Based Interventions

**DOI:** 10.3390/foods14152611

**Published:** 2025-07-25

**Authors:** Tianzhuo Huang, Huanzhi Lv, Yubo Lin, Xin Xiong, Yuqing Tan, Hui Hong, Yongkang Luo

**Affiliations:** 1 Beijing Laboratory for Food Quality and Safety, College of Food Science and Nutritional Engineering, China Agricultural University, Beijing 100083, Chinayuqingtan@cau.edu.cn (Y.T.);; 2 Zhejiang Guokuntang Health Holdings Group Co., Ltd., Hangzhou 310000, China

**Keywords:** *Cordyceps sinensis*, chilled storage, edible fungi preservation, energy metabolism

## Abstract

*Cordyceps sinensis* (*C. sinensis*) is widely recognized for its bioactive compounds and associated health benefits. However, due to its delicate nature, conventional chilled storage often results in the rapid degradation of valuable compounds, leading to loss of nutritional value and overall quality. This study integrated and evaluated comprehensive strategies: three gas-conditioning and two light-based preservation methods for maintaining both quality and nutritional integrity during 12-day chilled storage at 4 °C. The results revealed that vacuum packaging significantly inhibited weight loss (3.49%) compared to in the control group (10.77%) and preserved sensory quality (*p* < 0.05). UV-based interventions notably suppressed polyphenol oxidase and tyrosinase activities by 36.4% and 29.7%, respectively (*p* < 0.05). Modified atmosphere packaging (MAP) with 80% N_2_ and 20% CO_2_ (MAP-N_2_CO_2_) maintained higher levels of cordycepin (1.77 µg/g) and preserved energy charge above 0.7 throughout storage. The results suggest that MAP-based treatments are superior methods for the chilled storage of *C. sinensis*, with diverse advantages and their corresponding shelf lives associated with different gas compositions.

## 1. Introduction

**Figure 1:**
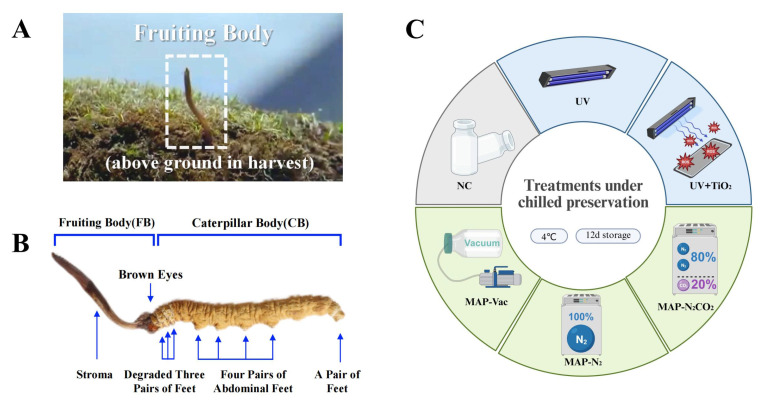
The structure and treatment methods of *C. sinensis*. (**A**) Growing and harvesting environment, (**B**) physiological structure, and (**C**) chilled storage treatments applied in this study. Note: NC: negative control group; UV: ultraviolet light irradiation; UV + TiO_2_: photocatalysis as ultraviolet light irradiated with the presence of TiO_2_; MAP-N_2_: modified atmosphere packaging with 100% N_2_; MAP-N_2_CO_2_: modified atmosphere packaging with 80% N_2_ and 20% CO_2_; and MAP-Vac: vacuum packaging sealed with air moved.

*Cordyceps sinensis* (*C. sinensis*) is a medicinal and edible fungus that forms a unique parasitic complex when the fungus *OphioCordyceps sinensis* infects the larvae of bat moths (Hepialidae). This results in a composite organism composed of the larval carcass (caterpillar body) and the fungal fruiting body (Figure 1A) [1]. For centuries, *C. sinensis* has been a precious ingredient in traditional Chinese medicine. In Asia, especially in China, *C. sinensis* has great economic significance. It is one of the most valuable medicinal fungi, and its price in the market often reaches an excessively high level. With the popularity of the health and wellness industry, *C. sinensis* is used in various dietary supplements, functional foods, and traditional medical products around the world. The unique combination of the rarity of *C. sinensis*, its recognized health benefits, and cultural significance makes it a commodity of significant economic importance both regionally and internationally. Nutritionally, the unique parasitic relationship and fungal metabolic processes give rise to a wide array of bioactive compounds, such as polysaccharides, nucleosides, sterols, fatty acids, amino acids, and peptides [1]. These active substances help *C. sinensis* to exert its distinctive nutritional profile and renowned health benefit properties (Figure 1B), such as immunomodulatory [2], anti-inflammatory, and antioxidative effects, hepatoprotective properties, and anti-aging potential [3].

Fresh *C. sinensis* contains the most diverse and bioactive compounds, providing significant nutritional and functional benefits. However, fresh *C. sinensis* is a precious seasonal product that only grows in high-altitude and snow-capped regions, and reaches optimal harvest time temporarily during April and May [4]. Like many fungal foods, fresh *C. sinensis* exhibits a high metabolic rate, is prone to enzymatic browning, and lacks protective barriers against microbial spoilage. As a result, fresh *C. sinensis* has a short shelf life of only seven days under chilled storage at 4 °C. This limited shelf life leads to significant losses in nutritional quality and also restricts its commercial distribution and acceptance. To ensure year-round market availability, *C. sinensis* is typically processed through drying and freezing, methods that alter its inherent form and quality. Traditional techniques such as hot air drying often lead to the degradation of sensitive compounds like cordycepin and flavonoids due to prolonged high-temperature exposure [5]. Although advanced approaches like freeze-drying offer better protection for heat-sensitive nutrients, they are costly and less feasible for large-scale or rapid commercial application. Similarly, conventional freezing slows enzymatic browning, but promotes the formation of large ice crystals, which can damage cellular structures and compromise texture. While improved methods such as rapid or cryogenic freezing help to reduce such damage, their reliance on specialized equipment limits their widespread adoption [4]. Given these limitations and considering that *C. sinensis* is highly susceptible to microbial spoilage, enzymatic browning, and oxidative degradation even under chilled conditions, there is an urgent need for a preservation method that can noticeably delay quality deterioration. An ideal approach should delay quality deterioration by minimizing oxygen exposure, inhibiting microbial and enzymatic activity, and maintaining the structural and nutritional integrity of fresh *C. sinensis* during short-term cold storage.

A chilled environment combined with modified atmosphere packaging (MAP) and light-based treatments has shown significant advantages in preserving perishable foods during refrigeration. MAP, which involves altering the gas composition around the product, has been successfully applied to slow down the respiration rate, limit microbial growth, and reduce moisture loss, thereby preserving a variety of perishable fungal foods’ freshness and quality [6,7,8,9]. Light-based treatments, particularly ultraviolet (UV) irradiation, have emerged as effective methods for microbial inactivation and enzymatic browning reduction in food products [10]. UV treatment works via the non-thermal disruption of the DNA of microorganisms, thereby inhibiting their growth and reproduction. The bactericidal effects of UV irradiation can be further enhanced through photocatalytic processes: when UV light irradiates semiconductor materials such as titanium dioxide (TiO_2_), reactive oxygen species (ROS) generation further contributes to the oxidative damage of microbial cell membranes, increasing the antimicrobial efficacy beyond that of UV irradiation alone [11].

This study aimed to evaluate the effectiveness of different preservation strategies in maintaining the post-harvest quality of *C. sinensis* during chilled storage (Figure 1C). These strategies included three gas-conditioning methods—vacuum packaging with complete air removal, modified atmosphere packaging using pure nitrogen, and a gas mixture composed of 80% nitrogen and 20% carbon dioxide—as well as two light-based interventions, involving ultraviolet irradiation and photocatalysis induced by UV light in the presence of titanium dioxide. The ultraviolet treatment was conducted at a wavelength of 254 nanometers for 15 min. We systematically assessed sensory traits, color, electrolyte leakage, and tissue structure to monitor quality deterioration. Nutritional components, including total sugars, free amino acids, and nucleosides, were quantified to track compositional changes. The antioxidant capacity was examined through the measurement of key enzyme activities and total antioxidant levels. These indicators were further analyzed in relation to changes in the bacterial community to explore the potential links between microbial succession and quality loss. By integrating physicochemical, biochemical, and microbial data, this work provides a comprehensive framework for understanding the spoilage process of *C. sinensis* and offers practical guidance for improving cold chain preservation techniques.

## 2. Materials and Methods

### 2.1. Materials

#### 2.1.1. Fresh *C. sinensis* Selection and Grouping

The fresh wild *C. sinensis* used in the experiment was supplied by the Guokuntang Co., Ltd. (Hangzhou, China). To ensure consistency across treatments, 400 intact and mold-free specimens with no visible damage were carefully selected. All samples originated from Yushu, Qinghai Province, and were harvested in June to ensure a uniform growth stage. The selected specimens had an average weight of 0.65 ± 0.05 g and an average length of 5.71 ± 0.42 cm. From harvesting through cleaning, selection, and cold chain transport (maintained at 4 ± 2 °C), to storage in the laboratory refrigerator, the entire process was completed within 48 h.

#### 2.1.2. *C. sinensis* Treatment

The *C. sinensis* samples were randomly divided into six groups, with each group subjected to different treatments as follows:

Negative control (NC) samples were directly packaged without any treatment and served as the control group. Modified atmosphere packaging with nitrogen (MAP-N_2_) samples were sealed in an atmosphere containing 100% nitrogen. Modified atmosphere packaging with nitrogen and carbon dioxide (MAP-N_2_CO_2_) samples were sealed in an atmosphere containing 80% nitrogen and 20% carbon dioxide. MAP-N_2_ and MAP-N_2_CO_2_ were conducted using gas-tight polypropylene preservation containers. These containers, commonly referred to as “fresh-lock boxes,” ensured minimal gas exchange during storage. After that, they were packaged through a semi-automatic box modified atmosphere packaging machine (DM-350B, Dajiang Machinery Equipment Co., Ltd., Nanjing, China). For the vacuum packaging (MAP-Vac) treatment, flexible food-grade plastic film bags were used to encase the samples prior to vacuum sealing. This approach was chosen to prevent physical damage or deformation, which may occur when rigid containers are subjected to high vacuum pressure. They were packaged and sealed using a vacuum sealer (No.14886, Deli Group Co., Ltd., Ningbo, China). Ultraviolet treatment (UV) samples were exposed to ultraviolet light for a 15 min duration at 254 nm before packaging. Photocatalysis treatment (UV + TiO_2_) samples were treated with ultraviolet light in the presence of TiO_2_ for a 15 min duration at 254 nm before packaging.

After packaging, all six groups of samples were stored at 4 °C in a refrigerator. At each sampling point (days 0, 3, 6, 9, and 12), quality assessments were performed in triplicate (*n* = 3), except for sensory evaluation (*n* = 10 panelists) and color measurements (*n* = 6 measurements per sample).

### 2.2. Methods

#### 2.2.1. Color Quality and Visual Image

Prior to imaging, each *C. sinensis* specimen was wiped dry and positioned in a consistent orientation to ensure standardization. The imaging system included a photo box (ZJSYP80CM, Shenzhen New Vision Photographic Equipment Co., Ltd., Shenzhen, China), an image acquisition device, LED light strips, and a background. Illumination was provided by LED strips containing 120 bulbs each, with a power output of 15 watts. Measurements were conducted under a D65 standard illuminant with a 90° observer angle. A green background was used to enhance visual contrast and clearly differentiate the *C. sinensis* body from the surrounding environment. Color changes during chilled storage were evaluated using a colorimeter (V2.0, Sanenshi Technology Co., Ltd., Shenzhen, China), using the method outlined by Li et al. with slight modifications [12]. The L* (lightness), a* (red–green), and b* (yellow–blue) values at six random points on the caterpillar body of *C. sinensis* were recorded. The total color difference (ΔE) was calculated as follows:

(1) $$∆E=\sqrt{{\left(L^{*}-L_{0}\right)}^{2}+{\left(a^{*}-a_{0}\right)}^{2}{+\left(b^{*}-b_{0}\right)}^{2}}$$

ΔE expresses the degree of color change in comparison with the initial color values of fresh *C. sinensis*. L_0_, a_0_, and b_0_ represent the color values of fresh *C. sinensis* on the starting day, and L*, a*, and b*, respectively, represent the color values measured at each time point after storage.

#### 2.2.2. Sensory Evaluation Method

All taste-related materials and instruments used in the study were food-grade to ensure participant safety. According to the guidelines of the Institutional Review Board of China Agricultural University, ethical approval was not required for this research. Nevertheless, all procedures were conducted in accordance with established protocols to safeguard participants’ rights and privacy throughout the study. These agreements include no mandatory participation, the ability to withdraw at any time, and the full disclosure of research requirements and risks. In addition, the written or oral consent of the participants was obtained, and the data of the participants was not released without their knowledge. Prior to participation, all panelists involved in the sensory evaluation provided informed consent. The assessment was carried out by a group of 10 individuals (five females and five males, aged 19 to 27) from the College of Food Science and Nutritional Engineering at China Agricultural University in Beijing, China. Sensory attributes and evaluation criteria were defined using a 10-point scale, as detailed in Table 1. For each sample, the final sensory score was calculated as the average of all individual ratings. A mean score below 6.0, representing less than 60% of the maximum, was defined as the sensory rejection threshold [13].

#### 2.2.3. Weight Loss Measurement

The weight of *C. sinensis* was measured before (M_0_) and after (M_1_) chilled storage to determine weight loss. Before weighing, any surface moisture was thoroughly removed using absorbent paper. Weight loss was then calculated using the following equation [14].

(2) $$Weight\;Loss\left(\%\right)=\left(M_{0}-M_{1}\right)/M_{0}\times 100$$

#### 2.2.4. Scanning Electron Microscopy (SEM)

Samples were sliced into 1 mm-thick sections and fixed in 2.5% glutaraldehyde solution (Cat No. P1126, Solarbio, Beijing, China). Prior to imaging, the tissues were dehydrated sequentially in ethanol solutions of 30%, 50%, 70%, 80%, 90%, and 100%, each for 30 min, followed by freeze-drying. The dried specimens were then sputter-coated with gold and observed under a scanning electron microscope (SU3500, HITACHI, Tokyo, Japan) at 1000× magnification.

#### 2.2.5. Malondialdehyde and Relative Electrolyte Leakage

The cell integrity was evaluated through the malondialdehyde (MDA) content, and determined following our previous study, with results expressed in μmol/g fresh weight [4]. The relative electrolyte leakage was assessed following the description of Feng et al. with editions: First, 0.5 g of samples was collected and placed in 20 mL of distilled water for 1 h at 25 °C to test the conductivity (C_1_) [15]. Then, the samples were boiled in a water bath. After cooling, the total conductivity (C_2_) was recorded at the same volume. The relative electrolytic leakage was calculated using the following equation:

(3) $$Relative\;electrolyte\;leakage\;rate(\%)=C_{1}/C_{2}\times 100$$

#### 2.2.6. Total Sugar, Amino Acid, and Nucleosides Content

The total sugar content (TSC) and amino acid content were measured using commercial assay kits (Cat No. BC2715 and BC1575, Solarbio, Beijing, China) following the manufacturer’s instructions. The concentrations of adenosine and cordycepin were quantified via HPLC, based on a method previously developed in our laboratory [4].

#### 2.2.7. Energy Status Assay

Adenosine triphosphate (ATP), adenosine diphosphate (ADP), and adenosine monophosphate (AMP) in *C. sinensis* tissue were assayed based on the protocol of the ATP, ADP, AMP content HPLC assay kit (Cat No. BC5114, Solarbio, Beijing, China). The ATP, ADP, and AMP contents were expressed as μg/g. The calculation method of the energy charge is referred to in the method of Feng et al., with the formula as follows [16]:

(4) $$Energy\;charge=\left(ATP+0.5\times ADP\right)/\left(ATP+ADP+AMP\right)$$

#### 2.2.8. Measurement of Superoxide Dismutase, Catalase, Tyrosinase, and Polyphenol Oxidase Activities

The enzymatic activities of superoxide dismutase (SOD), catalase (CAT), tyrosinase, and polyphenol oxidase (PPO) activities in *C. sinensis* were measured using commercial assay kits (Cat No. BC0175, BC0205, BC4055, BC0195, Solarbio, Beijing, China) following the manufacturer’s protocols. The enzyme activity levels were expressed as units per gram of fresh weight (U/g).

#### 2.2.9. Assay of Total Antioxidant Capacity

The total antioxidant capacity (T-AOC), representing the combined activity of various antioxidant compounds and enzymes, was measured using a commercial assay kit (BC1315, Solarbio, Beijing, China) according to the manufacturer’s instructions.

#### 2.2.10. Bacterial Community Analysis Method

The experimental design included two sampling timepoints: Baseline samples (day 0) were collected from fresh *C. sinensis* specimens, while six treatment groups were obtained after 12-day chilled storage. All biological specimens were aseptically packaged in sterile plastic bags following collection, cryopreserved on dry ice during transport, and delivered to the analytical laboratory within 30 min of collection to ensure sample integrity for subsequent bacterial community profiling. Microbial community genomic DNA was extracted, and after electrophoresis, the PCR products were purified using the AMPure^®^ PB beads (Pacific Biosciences, Menlo Park, CA, USA) and quantified with Qubit 4.0 (Thermo Fisher Scientific, Waltham, MA, USA). Equimolar amounts of purified PCR products were pooled for DNA library construction using the SMRTbell Prep Kit 3.0 (Pacific Biosciences, CA, USA). The resulting SMRTbell libraries were then sequenced on the Pacbio Sequel IIe System (Pacific Biosciences, CA, USA) by Majorbio Bio-Pharm Technology Co., Ltd. (Shanghai, China). The acquired raw data underwent bioinformatics analysis using the Majorbio Cloud platform for further analysis and interpretation [17].

#### 2.2.11. Statistical Analysis

All measurements were conducted three times, except for sensory analysis, which was carried out ten times, and color difference analysis, which was conducted six times. All measurement data are expressed as mean ± standard deviation. Statistical analysis was conducted using two-way ANOVA to evaluate the effects of treatment and storage time, followed by multiple comparisons. All analyses were performed using Windows GraphPad Prism 9 (Version 9.0.0, released in 2020; GraphPad Software, San Diego, CA, USA). Differences were considered statistically significant at *p* < 0.05. The Mantel test was processed through ChiPlot, and PCA was conducted using Windows GraphPad Prism 9.

## 3. Results

### 3.1. Apparent Quality Evaluation

#### 3.1.1. Color Quality and Difference

**Figure 2:**
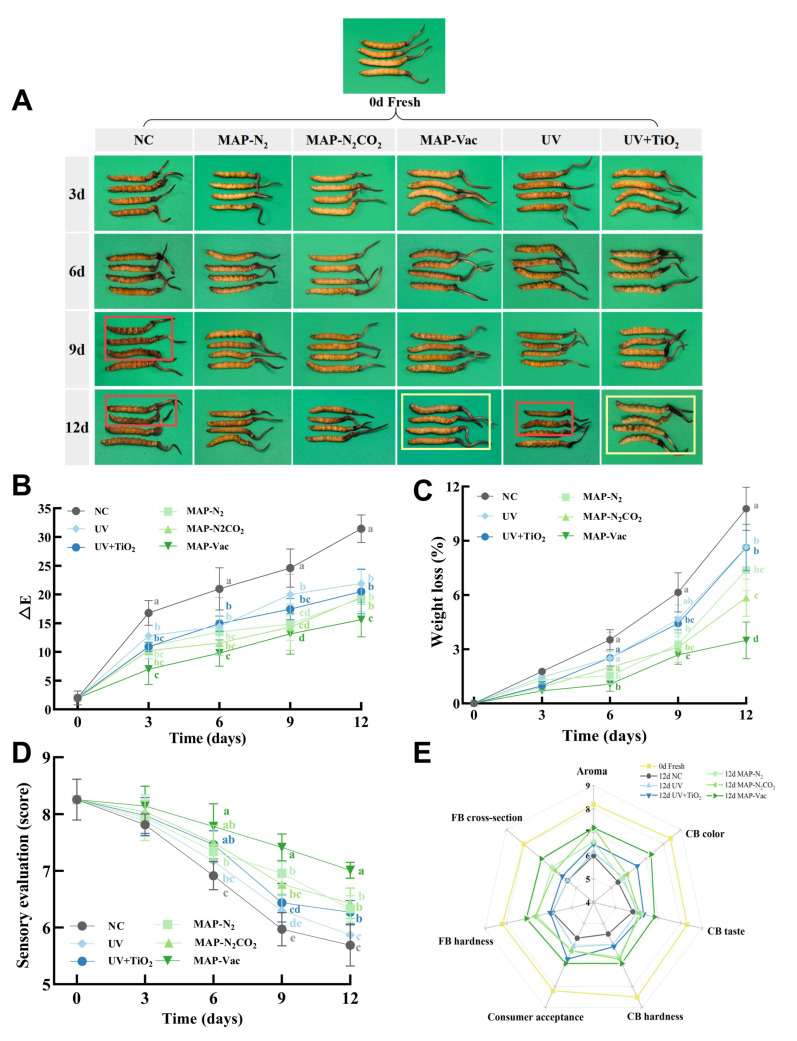
Changes in *C. sinensis*’s apparent quality: including (**A**) color quality, (**B**) color difference, (**C**) weight loss, (**D**) sensory evaluation, and (**E**) differences in sensory attribute ratings. The vertical bar represents the SDs of replicates; different letters indicate significant differences between treatment groups. Note: NC: negative control group; UV: ultraviolet light irradiation; UV + TiO_2_: photocatalysis as ultraviolet light irradiated with the presence of TiO_2_; MAP-N_2_: modified atmosphere packaging with 100% N_2_; MAP-N_2_CO_2_: modified atmosphere packaging with 80% N_2_ and 20% CO_2_; and MAP-Vac: vacuum packaging sealed with air moved.

Color is an important commercial quality indicator reflecting the chemical degradation and enzyme activity of *C. sinensis* [18]. The fresh *C. sinensis* was golden brown, well preserved, and showed no signs of deterioration (Figure 2A). The visual changes in *C. sinensis* over 12 days of chilled storage reveal significant differences between treatment groups. The dehydration of the NC group was aggravated, and visible discoloration and brown spots appeared in caterpillar body (CB) from day 9. UV + TiO_2_ and MAP-Vac maintained the original appearance over time, with the MAP-Vac group showing the slowest deterioration. During the enzymatic browning process, enzymatic reactions involving oxidases such as PPO require the participation of oxygen. Under vacuum treatment, the oxygen content is the lowest, so the degree of Browning is the smallest. TiO_2_ can effectively degrade phenolic compounds through photocatalysis, thereby reducing the occurrence of non-enzymatic browning reactions [19]. While the UV group showed some significant degradation compared with the UV + TiO_2_ group, this suggests that the photocatalysis treatment helps to slow down the browning process more effectively than UV alone.

In addition to the intuitive visual perception, the color changes in *C. sinensis* during storage can also be quantified through ∆E, indirectly reflecting the degree of browning and discoloration of *C. sinensis* (Figure 2B). The NC group has the largest increase in ∆E, indicating the most obvious browning, from golden yellow to dark brown. In contrast, on day 12, there were significant differences between the five treatment groups and the NC group (*p* < 0.05). However, only the MAP-Vac group exhibited significantly lower ∆E than all other treatment groups (*p* < 0.05), indicating superior color preservation performance. Consistent with the results of other studies, MAP effectively slowed down these processes by reducing the oxygen levels and limiting enzyme activity, thereby preserving the color of the *C. sinensis* [20]. This has also been demonstrated in the storage of other species. In the storage experiment on bayberry, the MAP treatment groups not only effectively inhibited the growth of microorganisms in color and delayed the browning process, but also significantly delayed the decline of soluble solids and ensured a high rate of good fruit [21].

#### 3.1.2. Sensory Evaluation

The sensory evaluation results, depicted through both sensory average scores (Figure 2D) and radar charts (Figure 2E), provide a comprehensive assessment of the sensory quality of *C. sinensis* under different preservation treatments during storage. As illustrated in Figure 2D, the significant differences between treatments happened from day 6. The sensory scores declined more rapidly during the latter period of storage, with pronounced divergence observed between the MAP-Vac and other treatments after day 9 (*p* < 0.05). This result is due to the fact that vacuum preservation can better lock in the moisture content of fresh *C. sinensis* (Figure 2C), allowing the water-soluble flavor substances of *C. sinensis* to be better retained. The unique salty and fresh taste of the fungal substances is released more thoroughly [22]. Due to the presence of moisture, *C. sinensis* stored under vacuum conditions becomes more golden and plumper. The other groups were all shriveled to varying degrees, which greatly reduced their edible evaluation.

The radar map (Figure 2E) further delineates sensory evaluations across seven dimensions: aroma, color, taste, fruiting body (FB), CB hardness, consumer acceptance, and cross-sectional characteristics. Compared to the NC group, the MAP-N_2_CO_2_ and MAP-Vac groups demonstrated superior performance in terms of aroma, hardness and cross-sectional indices. Among these, the MAP-Vac group significantly outperformed other treatment groups across all metrics. In contrast, the UV + TiO_2_ group, while performing better at the color and consumer acceptance levels, still lags behind MAP processing in most respects, although it helps to mitigate some of the texture degradation. The study of Sun et al. also demonstrated the multiple effects of MAP on maintaining the appearance, texture, and sensory qualities of Agaricus bisporus mushrooms under 5 °C storage conditions [6]. Confirmed in dairy products, MAP had a significant effect on shelf life extension by influencing the sensory, chemical, and microbiological properties during the refrigeration process [23].

#### 3.1.3. Weight Loss

In terms of weight loss (Figure 2C), all treatment groups exhibited progressive increases over time, with the NC group demonstrating the greatest weight loss of 10.77% by day 12. This trend aligns with the visual image of severe dehydration observations (Figure 2A). The MAP treatment groups exhibited slower weight loss rates, with the MAP-Vac and MAP-N_2_CO_2_ groups showing statistically distinct outcomes compared to other treatments by day 12 (*p* < 0.05). These results demonstrate that MAP approaches, particularly the MAP-Vac of 3.49%, effectively mitigate moisture loss in *C. sinensis* and preserve its structural integrity during storage. Light-based treatments also reduced weight loss compared with the NC group (*p* < 0.05), but their efficacy remained inferior to that of MAP-based methods.

### 3.2. Microstructure Observation and Cell Integrity

#### 3.2.1. *C. sinensis* Internal Hypha Structure

**Figure 3:**
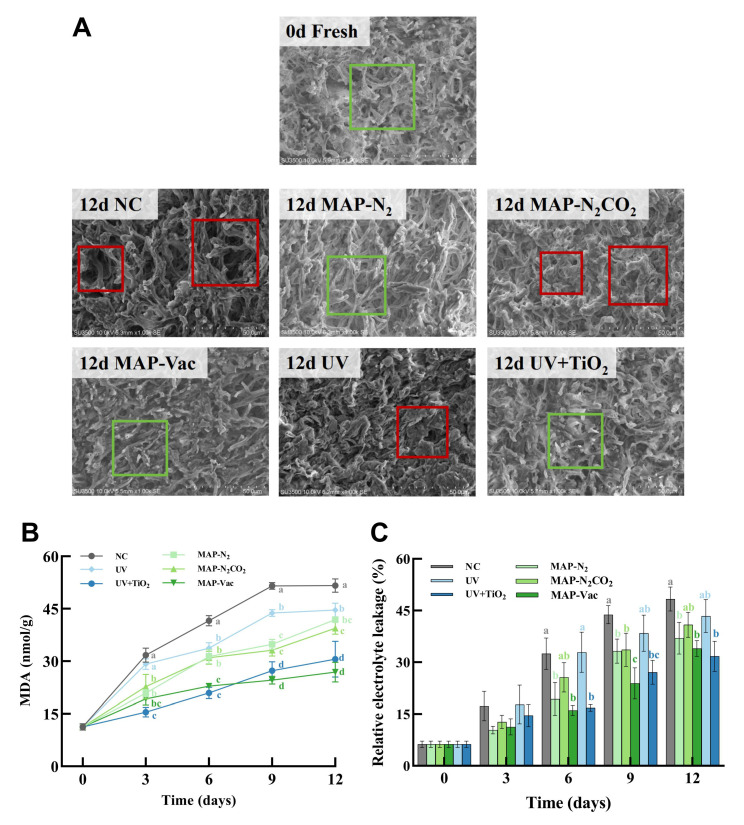
*C. sinensis*’s microstructure and cell integrity observation. (**A**) Microscopic images (SEM), (**B**) cell integrity (MDA), and (**C**) relative electrolyte leakage. The vertical bar represents the SDs of replicates; different letters indicate significant differences between treatment groups. Note: NC: negative control group; UV: ultraviolet light irradiation; UV + TiO_2_: photocatalysis as ultraviolet light irradiated with the presence of TiO_2_; MAP-N_2_: modified atmosphere packaging with 100% N_2_; MAP-N_2_CO_2_: modified atmosphere packaging with 80% N_2_ and 20% CO_2_; and MAP-Vac: vacuum packaging sealed with air moved.

In order to further study the changes in the hardness of *C. sinensis* under different storage conditions, we displayed the internal mycelial structure of *C. sinensis* from a microscopic perspective through SEM (Figure 3A). The surface of fresh mycelium is intact and presents a dense and evenly distributed reticular structure. Moreover, the mycelium surface is smooth without obvious damage or breakage, demonstrating good structural integrity. In the NC group, prominent structural degradation was observed, with the hyphal integrity clearly lost, leading to disorganization and fragmentation. Noticeable stacked structural deformation and mechanical injury were observed in UV and MAP-N_2_CO_2_ as well. In contrast, the MAP-Vac and UV + TiO_2_ groups maintained a better organized hyphal internal structure with less damage, suggesting the preserved cellular structure of *C. sinensis* [24], likely due to the vacuum environment and photocatalysis helping to slow down metabolic degradation. The hyphal structure difference between UV and UV + TiO_2_ highlighted the advantages of ROS in protecting the microstructure of *C. sinensis*.

#### 3.2.2. *C. sinensis* Hypha Cell Integrity

Figure 3B illustrates the variations in the malondialdehyde (MDA) content, a byproduct of the lipid peroxidation of cell membrane lipids representing membrane rupture and the loss of cell integrity; the elevated tendency is consistent with existing findings [4]. All five treatments exhibited MDA level inhibition compared to the NC group from day 6 (*p* < 0.05). Among these, the MAP-Vac and UV + TiO_2_ treatment demonstrated stable minimal MDA accumulation from day 6 (*p* < 0.05), demonstrating superior efficacy in mitigating oxidative stress and prolonging the storage quality of *C. sinensis*. SEM images (Figure 3A) further support that the cell structures in the MAP-Vac and UV + TiO_2_ were less damaged compared to in the NC group.

The relative electrolyte leakage (REL) is commonly measured to assess the degree of cell membrane damage and the leakage of cellular contents (Figure 3C), serving as an indicator of tissue integrity [16]. REL elevation is mechanistically linked to the disruption of membrane phospholipid bilayers and the subsequent efflux of intracellular electrolytes, thereby correlating with the extent of cellular lysis and degradation [25]. The REL values of all groups increased with the extension of refrigeration time, with the NC and UV groups exhibiting statistically significant hyperaccumulation of electrolytes compared with other groups (*p* < 0.05). At the endpoint, the REL of UV + TiO_2_, MAP-Vac, and MAP-N_2_ reached 31.70%, 33.98%, and 36.97%, respectively, prominent in protecting cell integrity. While MAP helps to prevent fungal foods by reducing oxygen exposure, UV + TiO_2_, compared with single UV irradiation, may have a more stabilizing effect on cellular membrane integrity. These results underscore the MAP-Vac and UV + TiO_2_ treatment in maintaining cellular integrity and reducing oxidative stress in edible fungi.

### 3.3. Antioxidant Capacity Evaluation

#### 3.3.1. Antioxidant Enzyme Activities

**Figure 4:**
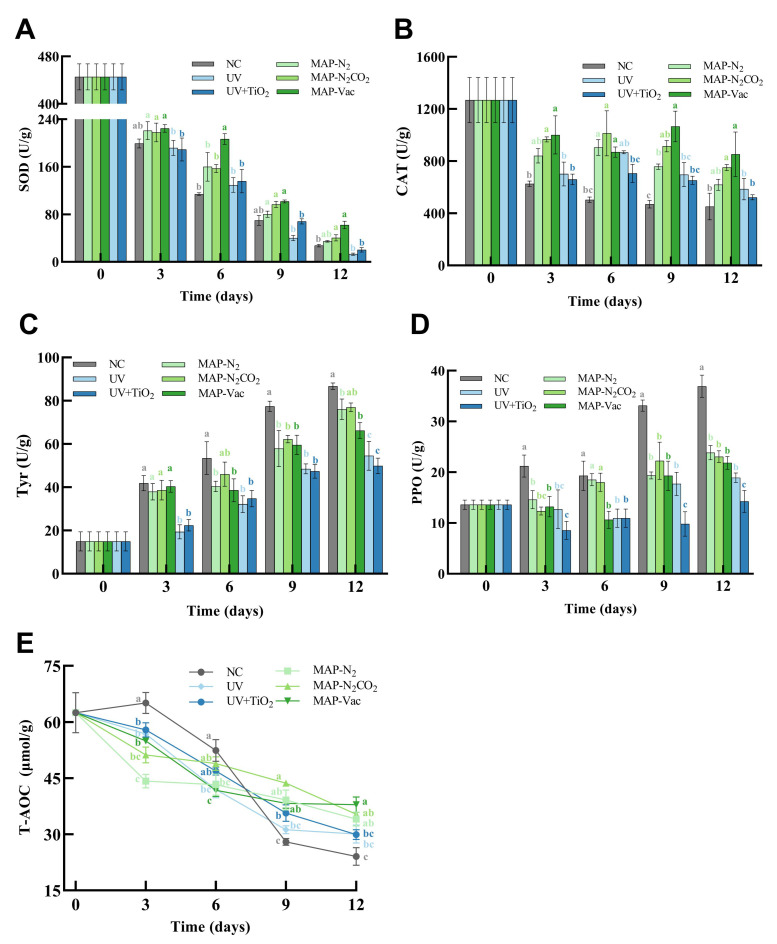
Changes in *C. sinensis*’s antioxidant capacity, including (**A**) SOD, (**B**) CAT, (**C**) Tyr, (**D**) PPO, and (**E**) T-AOC. The vertical bar represents the SDs of replicates; different letters indicate significant differences between treatment groups. Note: NC: negative control group; UV: ultraviolet light irradiation; UV + TiO_2_: photocatalysis as ultraviolet light irradiated with the presence of TiO_2_; MAP-N_2_: modified atmosphere packaging with 100% N_2_; MAP-N_2_CO_2_: modified atmosphere packaging with 80% N_2_ and 20% CO_2_; and MAP-Vac: vacuum packaging sealed with air moved.

As depicted in Figure 4A, the MAP treatments (MAP-N_2_, MAP-N_2_CO_2_, and MAP-Vac) generally exhibited higher SOD activity compared to the NC and light-based treatment groups (UV and UV + TiO_2_) at refrigeration endpoint. Notably, the MAP-Vac group showed effective SOD activity retention throughout the chilled storage period (*p* < 0.05), preserving the antioxidant capacity of *C. sinensis*. In contrast, the UV-based treatment group displayed no difference from the NC group in SOD activity, resulting from prolonged UV exposure-induced cellular damage that compromised enzymatic antioxidant defenses [26]. The catalase activity, shown in Figure 4B, followed similar trends to SOD activity. The MAP-Vac and MAP-N_2_CO_2_ treatments demonstrated the CAT activity level retention compared to the NC group from day 3 to day 12 (*p* < 0.05), highlighting their capacity to mitigate oxidative stress and preserve enzymatic antioxidant defenses during refrigerated storage. CO2-induced intracellular acidification has been reported to impair catalase tertiary structure stability, leading to a decrease in antioxidant enzyme activity [7]. The preservation of CAT functionality correlates with reduced lipid peroxidation markers as MDA (Figure 3B), suggesting that these treatments effectively counteract ROS accumulation. Similarly, the activity of antioxidant enzymes in mushrooms also decreases day by day during the refrigeration process. Moreover, with the help of oxygen isolation, the activity of antioxidant enzymes is well maintained [27]. These findings underscore that the MAP methods, particularly MAP-Vac, stand out as particularly effective strategies for preserving antioxidant enzyme activity, which is crucial for maintaining the health benefits of this edible fungus.

#### 3.3.2. Polyphenol Oxidase and Tyrosinase Enzyme Activities

Polyphenol oxidase and tyrosinase are pivotal enzymes in fungal food browning, mediating melanogenesis through distinct biochemical pathways. PPO facilitates melanin synthesis via phenolic oxidation, while tyrosinase catalyzes the hydroxylation of L-tyrosine to L-dopa, a critical melanin precursor. The regulation of these enzymatic activities is crucial for maintaining the organoleptic properties of *C. sinensis*, particularly color stability. As demonstrated in Figure 4C,D, both PPO and tyrosinase activities exhibited progressive upregulation during storage, correlating with accumulated oxidative stress (Figure 2B) and aligning with our former research [4].

As demonstrated in Figure 4C, the MAP-N_2_ and MAP-Vac treatments suppressed the increase in tyrosinase activity compared to the NC group from the middle stage of storage (day 6–12) (*p* < 0.05); UV-based treatments effectively inhibited the enzyme activity increase throughout the storage period (*p* < 0.05), making contributions of maintain the apparent qualities of *C. sinensis* by reducing the browning process. Figure 4D presents the PPO activity trends across different treatments, similar to tyrosinase activity. In the later period of chilled storage (day 9–12), all five treatments were effective in maintaining PPO activity levels (*p* < 0.05). Ali’s results also indicate that the absence of oxygen can reduce the activity of PPO enzymes, decrease the oxidation of phenolic substances, and minimize browning, thereby effectively inhibiting the maturation and aging of microorganisms, especially for UV + TiO_2_ treatment, which further supports the role of photocatalysis in preserving the color and sensory qualities [28]. Though both light-based interventions (UV and UV + TiO_2_) illustrated efficacy in inhibiting enzymatic browning, as shown in Figure 2A, the apparent color discoloration of UV and maintenance of UV + TiO_2_ need further mechanisms.

#### 3.3.3. Total Antioxidant Capacity

The total antioxidant capacity (T-AOC) varied across different treatments and storage periods (Figure 4E). The NC group exhibited a unique trend, with an increase in T-AOC during the early storage period (day 3–6), followed by a significant decline (*p* < 0.05) in the later stages (day 9–12). This initial increase without fresh-keeping intervention may be attributed to the rapid degradation of high-molecular-weight antioxidants into smaller, more reactive compounds, which temporarily boosts the overall antioxidant capacity. However, as storage continues, these smaller antioxidants are quickly consumed through oxidative reactions, leading to a significant drop in T-AOC.

In contrast, the MAP treatments demonstrated the steady maintenance of the T-AOC level, with significant high-level T-AOC at day 6–9 compared with the NC group (*p* < 0.05), indicating their effectiveness in preserving antioxidant properties in later chilled periods. The MAP-N_2_ and MAP-N_2_CO_2_ treatments exhibited preservation effects with no significant differences between them in the whole storage period, suggesting comparable preservation effects at this gas ratio for T-AOC. Another study also showed that the T-AOC content of fruits decreased over time during post-harvest refrigerated storage [29]. These findings indicate that, in the absence of preservation treatments, chilled storage may exacerbate the degradation of the antioxidant system in *C. sinensis*. This effect is likely associated with increased molecular mobility, which can elevate oxidative stress and promote the breakdown of sensitive biochemical constituents.

### 3.4. Nutritional Value Evaluation

#### 3.4.1. Dynamic Changes in Total Sugars and Total Amino Acid Content

**Figure 5:**
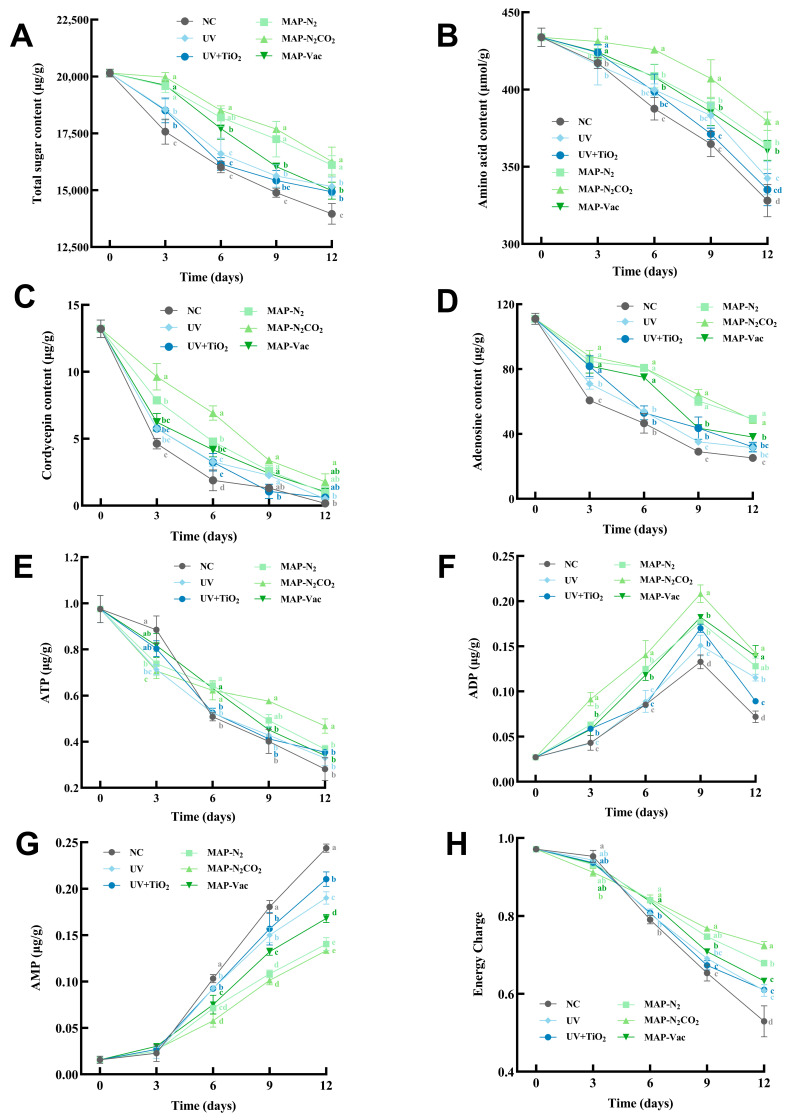
Changes in *C. sinensis*’s nutrients, including (**A**) total sugar content, (**B**) amino acid content, (**C**) cordycepin, (**D**) adenosine content, (**E**) ATP, (**F**) ADP, (**G**) AMP, and (**H**) energy charge. The vertical bar represents the SDs of replicates; different letters indicate significant differences between treatment groups. Note: NC: negative control group; UV: ultraviolet light irradiation; UV + TiO_2_: photocatalysis as ultraviolet light irradiated with the presence of TiO_2_; MAP-N_2_: modified atmosphere packaging with 100% N_2_; MAP-N_2_CO_2_: modified atmosphere packaging with 80% N_2_ and 20% CO_2_; and MAP-Vac: vacuum packaging sealed with air moved.

Figure 5A illustrates the temporal decline in the total sugar content (TSC) during refrigerated storage. The modified atmosphere packaging treatments demonstrated superior preservation efficacy compared to light-based interventions, especially inhibited carbohydrate degradation during the early storage period (*p* < 0.05). The total sugar content of MAP-N_2_ and MAP-N_2_CO_2_ was significantly higher than that in the MAP-Vac group from day 9 (*p* < 0.05). Longitudinal analysis revealed comparable sugar retention mechanisms in MAP-N_2_ and MAP-N_2_CO_2_, despite differing atmospheric compositions [30]. Figure 5B illustrates the progressive decline in the amino acid content of *C. sinensis* flesh subjected to six preservation methods, consistent with enzymatic hydrolysis and oxidative degradation during storage. The NC group displayed the steepest reduction, plummeting to 328.09 μmol/g by day 12, a 24.37% decline in the fresh status, underscoring the vulnerability of *C. sinensis* to unmitigated biochemical deterioration. Gas-conditioning approaches exhibited a gradual decline, significantly maintaining the amino acid concentration compared to in the NC group, whereas the MAP-N_2_CO_2_ group demonstrated superior preservation—379.49 μmol/g at the endpoint, with a 12.52% reduction. Despite the theoretically equal advantage of MAP treatments in oxygen exclusion, MAP-Vac has a marginally lower efficacy compared to MAP-N_2_CO_2_, suggesting that residual interstitial oxygen or physical compression effects may limit its performance, whereas CO_2_’s antimicrobial action provides additive protection. As carbohydrates and proteins constitute the major organic components of *C. sinensis* [31], Figure 5A,B revealed that MAP played a significant role in slowing nutrient degradation during chilled storage.

#### 3.4.2. Nucleosides Dynamics

Cordycepin and adenosine are the pivotal bioactive nucleosides in *C. sinensis*. The dietary intake of mushroom nucleosides regulates critical physiological processes [32] and serves as a functional biomarker for quality evaluation [33]. Against this backdrop, Figure 5C shows the degradation kinetics of cordycepin during chilled storage. While all groups showed degradation, MAP-N_2_CO_2_ maintained the highest cordycepin levels, declining from 13.21 µg/g to 1.77 µg/g, UV-based treatments showed limited utility, with the NC group exhibiting the steepest decline (0.16 µg/g), highlighting the imperative for active preservation strategies. MAP-N_2_CO_2_ was the most effective preservation method during the initial 6-day period (*p* < 0.05), attributable to its synergistic mechanisms: optimized gas composition (N_2_/CO_2_)-attenuated oxidative pathways, while microbial suppression curtailed enzymatic hydrolysis. These dual effects collectively stabilize nucleoside integrity, positioning MAP-N_2_CO_2_ as the optimal protocol for preserving cordycepin bioactivity in *C. sinensis*.

The adenosine content exhibited inherent instability and sensitivity to storage conditions (Figure 5D). Among all treatments in decline, MAP-N_2_CO_2_ and MAP-N_2_ maintained the highest adenosine level, outperforming MAP-Vac (*p* < 0.05). This divergence underscores the gas substitution preservation mechanism: hypha cell anaerobic respiration-induced intracellular acidification inhibits adenosine kinase, a key enzyme converting adenosine to inosine [34]. In contrast, the transient efficacy of MAP-Vac likely results from residual oxygen reactivating degradation pathways from day 9, despite initial oxygen exclusion. Notably, the NC group maintained the lowest adenosine level, a 77.29% loss compared with the fresh status, a phenomenon reported as microbial metabolism [4] in *C. sinensis* flesh, whereas UV-based treatments achieved partial mitigation through microbial inactivation, albeit insufficient to prevent significant degradation. Collectively, MAP-N_2_CO_2_ demonstrated the most prominent protective effect on nutrients of *C. sinensis* in the process of low-temperature storage. Overall, MAP processing groups exhibited superior protective effects compared to light-based treatments, and the MAP-N_2_CO_2_ group showed the best preservation effect in maintaining nutrient content.

#### 3.4.3. ATP, ADP, AMP, and Energy Charge

ATP, ADP, AMP, and energy charge constitute critical biomarkers of cellular metabolic vitality in *C. sinensis*. ATP serves as a key indicator of post-harvest energy metabolic activity, with its depletion correlating strongly with microbial proliferation and autolytic degradation [25,35]. Figure 5E illustrated the dynamic of ATP during chilled storage: at the endpoint, MAP-N_2_CO_2_ had 39.96% higher ATP levels than NC and was significantly different from all other groups. In contrast, MAP-N_2_ and MAP-Vac attenuated aerobic respiration, and their ATP retention remained limited due to persistent anaerobic microbial activity. The possible reason for the transient ATP surge observed in the NC group during the early stage is environment-induced stress. Preservation treatments like irradiation and gas conditioning may trigger an immediate cellular stress response in the treatment groups, accelerating ATP hydrolysis for the repair process. For example, UV exposure induced membrane damage, necessitating ATP-dependent lipid repair, whereas the MAP-N_2_CO_2_ leveraged CO_2_’s lipophilic properties to permeate cellular membranes, inducing intracellular acidification, which disrupted proton gradients and heightened ion balance demands on ATP [8]. By day 6, the NC group’s ATP reserves collapsed under uncontrolled microbial consumption and autolysis, whereas the treated groups stabilized due to active preservation mechanisms. These findings establish MAP-N_2_CO_2_ as the premier method for maintaining ATP pools in *C. sinensis*, synergistically balancing microbial suppression with cellular homeostasis.

Figure 5F illustrates the temporal dynamics of ADP levels in *C. sinensis* under refrigerated storage conditions across preservation treatments, peaking at day 9 after a decline. MAP-N_2_CO_2_ treatment results in the highest ADP levels throughout the storage period, peaking at 0.21 μg/g on day 9. The post-day 9 decline in ADP levels under MAP-N_2_CO_2_ treatment likely arises from a metabolic equilibrium shift and enzymatic feedback regulation. ADP, as an intermediate in ATP hydrolysis, accumulates when ATP is rapidly consumed. However, beyond day 9, two counteracting mechanisms dominate. Depleted ATP pools trigger adenylate kinase to convert two ADP molecules into ATP and AMP, reducing ADP reserves [36]. While CO_2_ initially suppresses spoilage microbes via intracellular acidification, prolonged storage may select acid-tolerant species that metabolize ADP as an alternative energy source. Intermediate ADP levels were observed in MAP-N_2_, MAP-Vac, UV, and UV + TiO_2_ treatments, characterized by temporal fluctuations. AMP accumulation during the chilled storage of *C. sinensis* reflects the interplay between endogenous enzymatic activity and microbial degradation (Figure 5G). The NC exhibited the highest AMP concentration (0.24 µg/g at day 12), consistent with unregulated ATP→ADP→AMP hydrolysis driven by residual mitochondrial activity and microbial phosphatases [37]. Previous studies indicate that Pseudomonas exacerbates post-harvest deterioration by enhancing respiratory metabolism and inducing energy deficits, exhibiting higher respiratory metabolism-related enzyme activity and lower ATPase activity, leading to diminished ATP/ADP levels and elevated AMP (Figure 5E,F) [38]. In contrast, MAP-N_2_CO_2_ limited AMP to 0.13 µg/g, a 50% reduction. The suppressed aerobic respiration thereby reduced ATP consumption and subsequent ADP hydrolysis to AMP.

Energy charge (Figure 5H) serves as a biochemical indicator of cell viability and metabolic integrity [39]. Initially, the NC group displayed a transient superiority in energy charge during the early storage phase (day 0–3), possibly due to residual ATP synthesis in freshly harvested tissues. The decline to 0.51 by day 12, primarily driven by microbial proliferation and associated enzymatic hydrolysis, corresponded with the depletion of nutrients and secondary metabolisms for energy metabolism. In contrast, MAP-N_2_CO_2_ demonstrated superior preservation of energy charge throughout the storage period (*p* < 0.05). The conservation above 0.7 can be attributed to the dual-action mechanism of CO_2_-induced intracellular acidification, which inactivates ATPases and microbial nucleotides, and the oxygen-displacing effect, which reduces oxidative degradation, aligns with its better preservation of carbohydrate and amino acid concentrations (Figure 5A,B). These findings highlight the unique advantage of MAP-N_2_CO_2_ in simultaneously targeting multiple degradation pathways, ensuring the more effective preservation of both energy charge and key metabolic substrates.

### 3.5. Bacterial Community Analysis

#### 3.5.1. Bacterial Community Compositions

**Figure 6:**
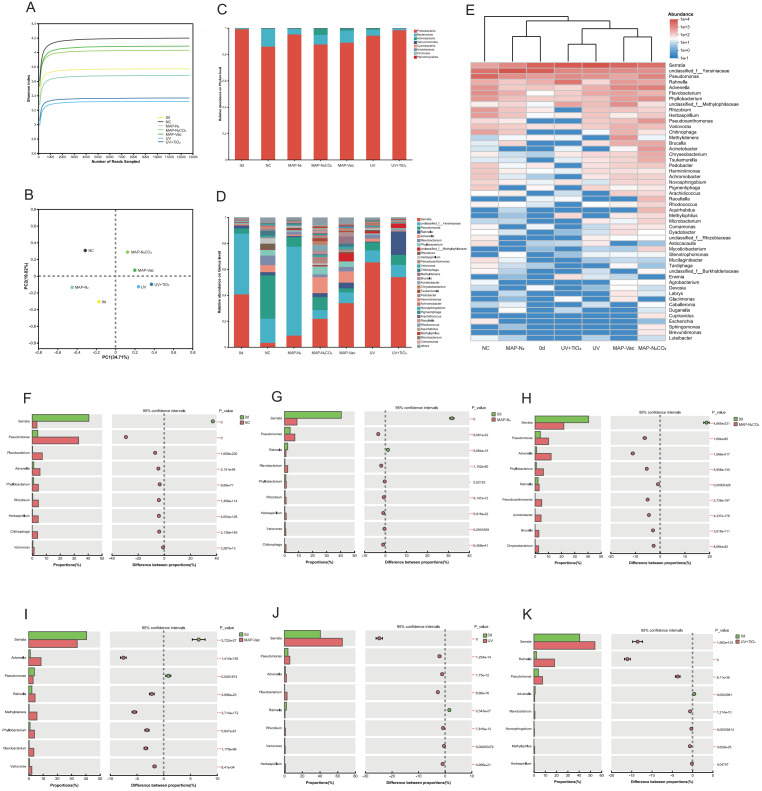
Changes in bacterial community: Shannon curves (**A**) and PCoA analysis (**B**) of different treatments of *C. sinensis* during chilled storage. Taxonomic distribution of bacterial communities at the phylum level (**C**) and genus level (**D**), and clustering heatmap at genus level (**E**). Fisher exact test bar plot of microorganisms at the genus level: 0 d vs. 12 d NC (**F**), 0 d vs. 12 d MAP-N_2_, (**G**), 0 d vs. 12 d MAP-N_2_CO_2_ (**H**), 0 d vs. 12 d MAP-Vac (**I**), 0 d vs. 12 d UV (**J**), and 0 d vs. 12 d UV + TiO_2_ (**K**). The 0 d represents fresh status at day 0 without chilled storage. Note: NC: negative control group; UV: ultraviolet light irradiation; UV + TiO_2_: photocatalysis as ultraviolet light irradiated with the presence of TiO_2_; MAP-N_2_: modified atmosphere packaging with 100% N_2_; MAP-N_2_CO_2_: modified atmosphere packaging with 80% N_2_ and 20% CO_2_; and MAP-Vac: vacuum packaging sealed with air moved.

To evaluate the diversity and reliability of the bacterial community in *C. sinensis* during refrigerated storage, the Shannon index was calculated. As shown in Figure 6A, the Shannon index values tended to stabilize with increasing sequencing numbers, indicating that the sequencing depth obtained was sufficient and reliable for further analysis. Principal coordinates analysis (PCoA) was performed to assess the variations in the bacterial community composition across different treatments. As depicted in Figure 6B, a distinct separation was observed among the NC, MAP-Vac, and other treatment groups, appearing in separate quadrants. This separation suggested significant differences in the bacterial community composition after 12 days of chilled storage.

To further investigate these variations, an in-depth analysis of community relative abundance was conducted at both the phylum and genus levels: At the phylum level (Figure 6C), *Proteobacteria* and *Bacteroidota* were identified as the two dominant phyla in *C. sinensis* during chilled storage. At the genus level (Figure 6D), the dominant bacteria in fresh samples were *Serratia* (40.56%) and unclassified *Yersiniaceae* (46.98%). In the NC group, the top three bacteria were *Pseudomonas* (33.23%), unclassified *Yersiniaceae* (18.71%), and *Flavobacterium* (7.23%). In MAP-N_2_, unclassified *Yersiniaceae* (68.53%), *Serratia* (8.88%), and *Pseudomonas* (7.37%) were the main bacteria. In MAP-N_2_CO_2_, the leading bacteria were *Serratia* (21.81%), *Advenella* (12.16%), *Pseudomonas* (10.19%), unclassified *Yersiniaceae* (6.64%), and *Phyllobacterium* (6.33%). In MAP-Vac, the predominant bacterial communities were *Serratia* (34.01%), *Advenella* (8.64%), and unclassified *Yersiniaceae* (8.20%). In the UV group, *Serratia* (65.33%) and unclassified *Yersiniaceae* (9.53%) were the main bacteria. In the UV + TiO_2_ group, the leading bacterial communities were *Serratia* (54.07%) and *Rahnella* (18.26%). In summary, *Serratia* and *Yersiniaceae* were the two primary genera during the chilled storage of *C. sinensis*. This finding aligned with the results of bacterial flora detections in mushroom (*Agaricus bisporus*) flesh reported by Hou et al., which exhibited a dominant genus for spoilage during storage [40].

#### 3.5.2. Spoilage-Associated Microbial Succession

Hierarchical clustering of the microbial community revealed three primary consortia across treatment groups (Figure 6E). Notably, the NC group formed a distinct clade separately from all preservation-treated samples, indicative of divergent spoilage trajectories in untreated systems. Light-based interventions (UV and UV + TiO_2_) clustered separately from other treatments, suggesting that the exposure to UV light selectively influenced the growth of certain bacterial communities—a phenomenon referred to as selection pressure induced by photochemical treatment. The fresh sample group and the MAP-N_2_ samples were adjacent on the phylogenetic tree, indicating that their bacterial profiles remained more similar to the original state before storage. These patterns are consistent with previous studies. Pogorzelska-Nowicka et al. demonstrated that modified atmosphere packaging with different oxygen concentrations significantly altered the dominant bacterial taxa and shelf life outcomes in stored button mushrooms. While Bhavya & Umesh Hebbar reported that pulsed light treatments selectively inhibited spoilage-related microbes in perishable produce by altering microbial succession patterns, our results extend these findings by showing that both MAP- and UV-based strategies not only reshape the microbial community structure of *C. sinensis*, but also distinctly reduce the relative abundance of spoilage-associated genera such as *Pseudomonas* and *Chitinophaga* over 12 days of chilled storage [41,42].

Taxonomic analysis revealed preservation-specific changes in microbial composition, with several spoilage-associated taxa showing marked increases in relative abundance. For example, in the NC group, *Pseudomonas* accounted for 33.23% of the total bacterial community after 12 days of storage, compared to only 2.41% in the fresh group. Similarly, *Chitinophaga* emerged exclusively in the NC group (2.73%), but was not detected in any MAP-treated or UV-treated samples. These genera suggest adaptive glycoside hydrolase production, a mechanism enabling polysaccharide metabolism [43]. UV + TiO_2_-induced *Advenella* suppression mirrors its sensitivity to photocatalytic hydroxyl radicals, disrupting quorum sensing-regulated proteolytic activity, which has been experimentally validated in meat systems [44].

Additionally, the Fisher exact test (Figure 6F–K) was employed to compare the differences in the bacterial community composition between fresh status and the other six treatment groups at the genus level. The comparisons revealed distinct patterns across different preservation methods. *Serratia* showed increased abundance in NC and MAP preservation-treated samples, indicating its potential proliferation compared to fresh samples. Conversely, light-based treatments (UV, UV + TiO_2_) underwent a decrease during the storage process. Conversely, *Pseudomonas* exhibited a significant proliferation across all treatments, with the notable exception of MAP-Vac (*p* < 0.05), demonstrating the selective inhibitory effect of vacuum conditioning, potentially attributable to oxygen concentration-mediated metabolic suppression.

MAP-N_2_CO_2_ and UV + TiO_2_ treatments exhibited unique profiles. MAP-N_2_CO_2_ samples showed a notable increase in *Advenella*, implying that the specific gas composition creates an environment conducive to this genus. UV + TiO_2_ treatment led to a significant rise in *Rahnella*, highlighting the specific selection pressures exerted by this photochemical treatment. These findings collectively demonstrate that different preservation methods significantly alter the bacterial community composition in *C. sinensis* during refrigerated storage. The variations observed in the abundance of specific bacterial genera suggest that each preservation technique creates unique microenvironmental conditions that favor the growth of particular taxa.

### 3.6. Correlation and Difference Analysis

**Figure 7:**
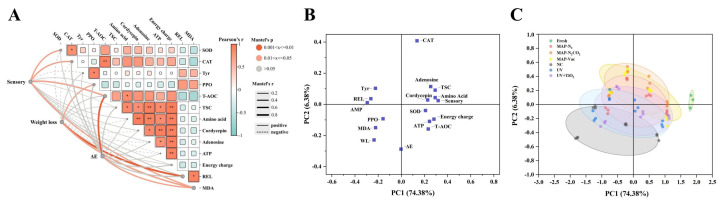
Mantel test and principal component analysis (PCA) of the overall quality of *C. sinensis*: (**A**) correlation analysis between apparent quality traits and biochemical indicators, (**B**) loading plot illustrating the association between quality indices and principal components, and (**C**) score plot illustrating the separation among different treatments. Note: Tyr: tyrosine; NC: negative control group; UV: ultraviolet light irradiation; UV + TiO_2_: photocatalysis as ultraviolet light irradiated with the presence of TiO_2_; MAP-N_2_: modified atmosphere packaging with 100% N_2_; MAP-N_2_CO_2_: modified atmosphere packaging with 80% N_2_ and 20% CO_2_; and MAP-Vac: vacuum packaging sealed with air moved. * indicates *p* < 0.05, ** indicates *p* < 0.01.

The correlation between the apparent quality and other indices of *C. sinensis* was analyzed using the Mantel test (Figure 7A). Apparent quality indices including color, sensory values, and weight loss are key determinants of consumer acceptance and market value [18]. Identifying significant biochemical indices linked to these quality indicators is beneficial for establishing a quality evaluation system for *C. sinensis*. Among the indices, SOD, CAT, PPO, T-AOC, REL, and MDA were significantly correlated with various apparent quality indicators. MDA showed a significant correlation with all the apparent quality indicators, suggesting that oxidative stress plays a central role in the degradation of *C. sinensis* during storage. This implies that MDA is a reliable indicator of quality degradation. Additionally, T-AOC was correlated with color and sensory value, highlighting the importance of oxidative damage in the quality deterioration of *C. sinensis* during chilled storage.

Principal component analysis (PCA) (Figure 7B,C) was performed to evaluate the effects of different preservation treatments on the quality of *C. sinensis* during chilled storage. The first two principal components (PC1 and PC2) explained 74.38% and 6.38% of the total variance, respectively. As shown in the loading plot (Figure 7B), several quality-related indices were closely linked to PC1. Parameters such as sensory score, total sugar content, adenosine, and amino acid content, which represent metabolism and apparent properties, exhibited a positive correlation with PC1, suggesting that these attributes were better maintained under optimal preservation conditions. Conversely, parameters including AMP, REL, PPO, and MDA showed negative correlations with PC1, implying that these markers of oxidative stress and metabolism degradation became more pronounced under suboptimal chilled treatments, potentially leading to quality degradation.

The score plot (Figure 7C) reveals a clear distinction between the fresh group and all chilled samples at the end of storage, with the fresh samples forming a tightly clustered group that is clearly separated from the others. Notably, the NC group was positioned farthest from the fresh samples, underscoring the deterioration in quality without preservation intervention and reinforcing the necessity for effective storage strategies. Among the treatment groups, MAP-Vac and MAP-N_2_CO_2_ displayed noticeable separation from the NC group and were positioned closer to the fresh sample cluster, indicating their superior effectiveness in preserving the overall quality of *C. sinensis* during chilled storage and their potential for broader application.

## 4. Conclusions

This study comprehensively evaluated various preservation methods for maintaining the quality, antioxidant capacity, and nutritional value of *C. sinensis* during chilled storage. The results demonstrated that MAP-N_2_CO_2_ and MAP-Vac were comprehensive, effective methods, consistently maintaining the overall quality and nutritional integrity of *C. sinensis* throughout the storage period, while UV-based treatments illustrated significant efficacy from their bactericidal effects and inhibiting browning enzymatic activities. Future research could explore combining the metabolic degradation inhibition of MAP with the sterilization effects of UV irradiation or photocatalysis in a sequential process to further extend shelf life. Additionally, investigating the effects of these combined methods on both quality retention and microbial community dynamics would provide deeper insights into optimizing storage protocols for *C. sinensis* and other perishable fungal products.

## Figures and Tables

**Table 1:** *Cordyceps sinensis* sensory evaluation standard.

Score	Aroma (S1)	CB Hardness (S2)	CB Color (S3)	CB Taste (S4)	FB Hardness (S5)	FB Cross Section (S6)	Consumer Acceptance (S7)	Sensory Score
10–8	Pleasant mushroom-like aroma	Very firm	Yellowish- white to golden-yellow	Sweet taste, lasting flavor	Very firm	Full of white content	Very satisfied	-
8–6	Mild mushroom-like aroma	Firm, less springy	Golden-yellow	Sweet taste, slightly weak and short-lived flavor	Firm, less springy	Quite full of white content	Satisfied	-
6–4	Mild and Neutral	Moderately firm	Brownish- yellow	Neutral and no off flavor	Moderately firm	Somewhat lacking white content	Acceptable	-
4–2	Slight off smell	Soft, lacks firmness	Tawny yellow	Bland or slightly off flavor	Soft, lacks firmness	Less white content	Unacceptable	-
2–0	Unpleasant, off odor	Very soft	Dark brown	Obvious off flavor	Very soft	No white content	Offensive	-

Note: Sensory score = average (S1, S2, S3, S4, S5, S6, and S7); CB: caterpillar body; and FB: fruiting body. The sensory rejection point of *C. sinensis* was judged by a sensory score of less than 6.0 (<60% of the full score).

## Data Availability

The original contributions presented in the study are included in the article, further inquiries can be directed to the corresponding author.

## Abbreviations

The following abbreviations are used in this manuscript:

ADP	Adenosine diphosphate
AMP	Adenosine monophosphate
ATP	Adenosine triphosphate
CAT	Catalase
CB	Caterpillar body
*C. sinensis*	*Cordyceps sinensis*
FB	Fruiting body
MDA	Malondialdehyde
MAP	Modified atmosphere packaging
MAP-N_2_	Modified atmosphere packaging with nitrogen
MAP-N_2_CO_2_	Modified atmosphere packaging with nitrogen and carbon dioxide
MAP-Vac	Vacuum packaging
NC	Negative control
PCA	Principal component analysis
PCoA	Principal coordinates analysis
PPO	Polyphenol oxidase
REL	Relative electrolyte leakage
ROS	Reactive oxygen species
SEM	Scanning electron microscopy
SOD	Superoxide dismutase
T-AOC	Total antioxidant capacity
TiO_2_	Titanium dioxide
TSC	Total sugar content
UV	Ultraviolet treatment
UV + TiO_2_	Photocatalysis treatment
